# Knowledge, attitudes, and practices regarding cervical cancer screening among women in metropolitan Lima, Peru: a cross-sectional study

**DOI:** 10.1186/s12905-021-01431-0

**Published:** 2021-08-18

**Authors:** Michelle M. Pieters, Rae Jean Proeschold-Bell, Emily Coffey, Megan J. Huchko, Lavanya Vasudevan

**Affiliations:** 1 Duke Global Health Institute, Durham, NC USA; 2 Center for Health Policy and Inequalities Research, Duke University, Durham, NC USA; 3 Duke University, Durham, NC USA; 4 Department of Obstetrics and Gynecology, Duke School of Medicine, Durham, NC USA; 5 Department of Family Medicine and Community Health, Duke School of Medicine, Durham, NC USA

**Keywords:** Cervical cancer, Cervical cancer screening, Health belief model, Peru, Low- and middle-income countries, Knowledge, attitudes and practices

## Abstract

**Background:**

Cervical cancer is a leading cause of cancer deaths among women of reproductive age in Peru. Screening and early identification of pre-cancerous lesions are a cornerstone of the cervical cancer prevention strategy. Yet, there is limited literature on barriers to screening among Peruvian women. In this cross-sectional study, we aimed to examine Peruvian women’s knowledge, attitudes, and practices regarding cervical cancer screening and identify possible reasons for the gap between knowledge and screening.

**Methods:**

The study was conducted in metropolitan Lima from June–August 2019. We purposefully recruited 12 women who had previously been screened, and 12 who had never been screened for cervical cancer. The women completed a 40-question knowledge and attitude survey and an in-depth interview about barriers to screening. Descriptive analysis was used to calculate a knowledge and attitude score and qualitative analysis was guided by the Health Belief Model constructs.

**Results:**

Previously screened participants had greater knowledge of cervical cancer symptoms, risk factors, and prevention (mean score = 28.08, S.D. = 4.18) compared to participants who had never been screened (mean score = 21.25, S.D. = 6.35). Both groups described lack of priority and embarrassment as barriers to cervical cancer screening. For participants who had never been screened before, major barriers included the fear of a cancer diagnosis and lack of information about screening services. Pregnancy, unusual gynecological symptoms and encouragement from friends and family were cues to action for participants seeking screening. Most participants in both groups recognized the benefits of getting screened for cervical cancer. Being previously screened increased participants’ self-efficacy for engaging in screening behaviors again. Misconceptions regarding screening procedures and cervical cancer were also noted as barriers for participants accessing screening services.

**Conclusions:**

Improving knowledge and awareness about cervical cancer and screening programs may improve screening behaviors among women. Targeting women who have never been screened before and addressing their fears and concerns around embarrassment may be other areas for intervention. Misconceptions that deter women from screening services are an important issue that should be addressed in order to increase the number of women who get timely screenings.

**Supplementary Information:**

The online version contains supplementary material available at 10.1186/s12905-021-01431-0.

## Background

Every five hours, a woman dies due to cervical cancer in Peru [1]. The incidence rate of cervical cancer in Peru is 25.2 per 100,000 women per year, which is much higher than the incidence rate of cervical cancer in South America at 18.2 per 100,000 per year [2]. Moreover, cervical cancer disproportionately impacts women in low-income neighborhoods and those living in rural and remote regions in Peru [3]. In an effort to reduce cervical cancer incidence and mortality in Peru, the Peruvian Ministry of Health (MINSA) recommends that sexually active women between the ages of 30–59 get screened for cervical cancer every three years [4]. Despite this proactive agenda for the prevention of cervical cancer incidence and mortality in Peru, recent studies suggest that only 53.9% of women in the target age groups are getting screened every three years as recommended [2]. Several barriers to women’s uptake of cervical cancer screening have been identified in the literature [1]. Current known barriers include low awareness of screening among women, embarrassment of being screened, and fear that screening results may reveal cancer [5]. However, additional research is needed on the interplay of these barriers and how they can be modified to achieve positive behaviors, i.e., uptake of preventive screenings.

The Health Belief Model (HBM) is a conceptual framework that has been widely used in health behavior research and health behavior interventions [6]. The constructs of this model include perceived susceptibility, perceived severity, perceived benefits, perceived barriers, cues to action, and perceived self-efficacy. The HBM posits that individuals are more likely to take action on a health behavior if they think they are susceptible to a condition, believe that the condition has severe consequences, believe that taking action will be beneficial in reducing that severity and susceptibility, and believe that the benefits of the action will outweigh the barriers [6]. Demographic factors can indirectly affect the perception of the constructs of the model. The constructs of the HBM have been found to explain participation in cervical cancer screening in Latina immigrants [7]. Additionally, specific cervical cancer interventions tailored to the model have been found to be effective [7]. Culturally relevant and theory-based interventions are necessary in the development of effective cervical cancer interventions. The HBM constructs, specifically perceived barriers, allow one to explore the reasons why women do not use preventative health services, in this case cervical cancer screening [7]. The HBM constructs are easy to use, and therefore have been used in several community-based interventions targeting vulnerable populations [8–10].

Previous studies have explored the knowledge, attitudes and practices (KAP) related to cervical cancer and cervical cancer related screening procedures in several countries, but there is limited literature on knowledge, attitudes, and practices in Lima, Peru. To date, few qualitative studies in Peru have examined barriers from a woman’s perspective and only a small number of studies have examined perceptions of women who have never been screened for cervical cancer [1, 11]. Understanding women’s experiences could be helpful in uncovering factors that facilitate or prevent women from getting screened. Furthermore, examining the knowledge, attitudes, and practices in relation to cervical cancer screening procedures can establish a baseline for use in future assessments and help measure the effectiveness of interventions to change health-related behaviors. The aims of the study were to: examine women’s knowledge, attitudes and practices regarding cervical cancer screening, its risk factors, and prevention methods using components of a relevant health behavior theory, namely the Health Belief Model and to identify possible reasons for the gap between knowledge and screening.

## Methods

### Study design

From June–August 2019, we conducted a cross-sectional study, with a single data collection event per participant, comprised of a survey and an in-depth interview with women in metropolitan Lima, Peru to understand their knowledge, attitudes, and practices related to cervical cancer screening.

### Study setting and partnership

The study was conducted in partnership with *Liga Contra el Cancer* (League Against Cancer), a non-profit private healthcare organization in Peru that aims to prevent cancer through awareness campaigns and cancer screening. *Liga Contra el Cancer* (La Liga) has five mobile community outreach units (COUs) that travel around low-income districts of metropolitan Lima to offer free cancer screening to women. These screening sessions include visual inspections using acetic acid (VIA) and Pap smears for cervical cancer, breast exams, rectal exams, and thyroid exams. Additional information about study setting and partnership is described in Additional file 1: File S1.

### Sampling strategy, sample size, recruitment, and participants

We used a purposive sampling approach and targeted recruitment in those districts in metropolitan Lima where the COUs were providing services during the study period. A total sample size of 24 participants was chosen based on publications in the literature, which suggest that 88% thematic saturation in qualitative studies can be achieved with 12–16 interviews [12]. Participants were grouped into two groups based on screening status: women who had previously been screened (n = 12) and women who had never been previously screened (n = 12). Participants with previous cervical cancer screening experience were recruited at the COUs before or after their appointment at the COU. Participants without cervical cancer screening experience were recruited from a variety of community settings near the COUs (e.g., markets, schools, public parks, and municipalities). Community health workers and health promoters from La Liga aided with recruitment. Recruitment was completed once 12 women were interviewed for each group. Participants were approached in person and told about the study. If they were interested, a screening questionnaire was administered to assess for eligibility and allocate to the study group (i.e., previously screened or not previously screened). We did not hold constant possible confounding factors in recruiting the two groups.

### Eligibility

Although MINSA recommends women who are sexually active between the ages of 30–49 be prioritized for cervical cancer, La Liga promotes cervical cancer screening to start when women initiate sexual activity. Hence, the study included women younger than 30 years of age. Since the risk of cervical cancer continues at older ages, we included women up to the age of 65 [13].

Women were eligible to participate in the study if they met the following criteria:

- Age 18–64 years.
- No history of hysterectomy.
- Able to provide informed consent for participation, including consent to have the interview audio-recorded.
- For group 1, prior experience being screened for cervical cancer or presentation to COU for screening.
- For group 2, no prior experience being screened for cervical cancer.

### Data collection

Following written informed consent, participants individually took part in a single data collection event that included two data collection components: a structured survey and an in-depth interview. All data collection was conducted in private tents that *La Liga* had set up next to the COUs. The structured survey took approximately 20 min, and the in-depth interview took approximately 20–30 min, and both were verbally administered by an interviewer in Spanish. Participants in the study were given PEN 10 (~ $5) to compensate for their time participating in the study. Participants were informed about compensation during the informed consent process.

The structured survey assessed knowledge, attitudes, and practices regarding cervical cancer risk factors, symptoms, and prevention. The survey included demographic questions such as educational and marital status, food security, parity, and age at first birth. Other questions were identified from existing knowledge, attitudes, and practices studies and adapted by the study team to the Peruvian context (See Additional file 1: File S2 for survey guide) [14, 15]. Overall, the structured survey included 40 cervical cancer knowledge-related multiple-choice questions, true/false questions on HPV knowledge, agree/disagree statements on community cervical cancer perceptions, and free-response questions on cervical cancer screening procedures.

The in-depth interview followed a guide, developed for this study, that included nine open-ended questions based on the Health Belief Model to examine women’s perceived susceptibility, severity, self-efficacy, cues to action, and barriers regarding cervical cancer and screening procedures (See Additional file 1: File S3 for interview questions) [6]. Each question was followed by a probe to elicit more information from the participant. There were two versions of the interview guide. One version was used for the participants who had previously been screened, and another version was tailored for participants who had never been screened.

### Data analysis

All categorical socio-demographic characteristics (education, employment, age, schooling, and marital status) measured in the survey were presented as frequencies and percentages, and continuous reproductive characteristics (number of births, age at first marriage, and age at first child) were summarized using their median values and range.

Overall, there were 40 questions in the survey; if a participant answered all questions correctly, she was awarded 40 points. Correct responses received a score of 1 while incorrect responses received a score of 0. A total knowledge and attitude score was computed individually for each participant. Total knowledge and attitude scores were summed by participant group (screened or not screened) and a mean score was computed for each group. The total of 40 points was derived from the survey sections, in which 32 points were attributed to knowledge and eight points to attitudes. We developed an ad-hoc classification schema based on tertiles of the knowledge and attitude score [23]. Respondents who had a score above 27 were considered as having high knowledge, while scores of between 14 and 26 points were considered as having a fair knowledge level. A score below 13 was considered as having low knowledge. The number of correct answers was then compared between participants who had never been screened and participants who had previous screening experience. We looked at the difference between the two groups using the Mann–Whitney U test. We used a Mann–Whitney U test because we wanted to find the difference of means between two small groups.

For analysis of the in-depth interviews, transcripts were uploaded to NVivo version 12 [16]. An applied thematic analysis approach was used to analyze implicit and explicit ideas through identifying key themes in the text, and for mapping data onto the theoretical framework of the Health Belief Model. An initial codebook was drafted following structural codes modeled after the HBM constructs. Three additional codes (Misconceptions, La Liga services, and Miscellaneous) were added to the codebook to capture themes not classified under the HBM. After drafting the initial codebook, two reviewers independently coded 20% of the transcripts and met to discuss and reach consensus on the coding definitions and application of codes. The codebook was updated based on the discussions. After all transcripts were coded, one researcher wrote analytic memos about each coding report, summarizing key themes across interviews. We looked at differences in themes between participants who had been screened and participants who had not been screened to look for discordant and concordant ideas. The themes were organized by HBM constructs and presented in summary form in a table as well as a narrative form in the text.

## Results

All but one participant approached for the study consented to participate. The reason for non-participation was lack of time.

Participants’ demographic characteristics are presented in Additional file 1: File S4. In general, participants ranged from 18 to 64 years of age. The majority of participants were between 21 and 49 years of age. Approximately two-thirds of participants were married or in a union. Three-quarters of participants reported completing secondary education, and the same proportion of participants were unemployed. The average age for getting married or starting to live with a partner was 22 years old and the average age of having a first child was 21.3 years old. Differences between sub-groups of participants were also evident. Participants who had never been screened were younger, had lower formal education, were more likely to be single and have lower number of births and were unemployed compared to those who had been screened before.

Table 1 shows respondents’ knowledge towards cervical cancer, Pap smears, and HPV. All participants knew that cervical cancer could be treated, but fewer (33.3%) participants knew what cervical cancer was. Most participants who had been previously been screened knew what a Pap smear was compared to only 16.7% of participants who had never been screened. 66.7% of participants who had been screened knew what HPV was compared to only one participant who had never been screened.

**Table 1:** Comparing knowledge of cervical cancer and HPV among participants who have been previously screened (n = 12) and participants who have never been screened (n = 12)

Variable	Previously screened (n = 12) n (%)	Never screened (n = 12) n (%)	Total (n = 24) n (%)
Knowledge of cervical cancer	6 (50)	2 (16.7)	8 (33.3)
Knowledge of Pap smears	11 (91.7)	2 (16.7)	13 (54.2)
Knowledge of HPV	8 (66.7)	1 (8.3)	9 (37.5)
Knowledge that HPV is spread by sexual contact	11 (91.7)	8 (66.7)	19 (79.2)
Knowledge of getting screened once a year	7 (58.3)	6 (50)	13 (54.2)
Knowledge that women with more sexual partners are predisposed to cervical cancer	12 (100)	9 (75)	21 (87.5)
Knowledge of cervical cancer prevention	12 (100)	9 (75)	21 (87.5)
Knowledge that not using condoms is a risk factor	12 (100)	11 (91.7)	23 (95.8)
Knowledge that having multiple partners is a risk factor	11 (91.7)	10 (83.3)	21 (87.5)
Knowledge of cervical cancer treatment	12 (100)	12 (100)	24 (100)
Footnotes: No missing data			

Table 2 shows the distribution of participants by knowledge and attitude score tertile. Overall, participants who had been screened had higher knowledge of cervical cancer (mean score = 28.08, SD = 4.18, range = 19–35) compared to participants who had never been screened (mean score = 21.25, SD = 6.35, range = 11–33). A Mann–Whitney U test indicated that the knowledge and attitude score for participants with previous screening experience was significantly higher than the knowledge and attitude score for participants with no previous screening experience (U = 24.5, *p* = 0.007).

**Table 2:** Knowledge scores

Variable	Previously screened (n = 12) n (%)	Never screened (n = 12) n (%)	Total (n = 24) n (%)
Low knowledge score	0 (0)	1 (8.3)	1 (4.2)
Fair knowledge score	2 (16.7)	9 (75)	11 (45.8)
High knowledge score	10 (83.3)	2 (16.7)	12 (50)
Footnotes: No missing data			

Data from the in-depth interviews revealed participants’ perspectives and experiences that were mapped onto the Health Belief Model. In total, we identified 23 themes and grouped them under the 6 constructs of the HBM. The key themes that emerged for each of the HBM constructs are summarized in Table 3 and described below.

**Table 3:** Health Belief Model as applied to cervical cancer screening

HBM construct	Overall themes	Concordant themes	Discordant themes
Perceived barriers	Lack of time to get screened for cervical cancer Fear of procedure/diagnosis of cervical cancer Low priority Embarrassment at being examined by a physician Lack of information regarding cervical cancer and cervical cancer screening procedures Lack of health insurance to cover cervical cancer screening Having a male examiner*	Low priority for screening Embarrassment	*Screened women:* Lack of time Lack of health insurance *Unscreened women:* Fear Lack of information
Cues to action	Outside personal influences (family, friends, partners) Previous pregnancy Presenting with unusual gynecological symptoms	Family encouragement Presenting with gynecological symptoms	*Screened women:* Pregnancy
Perceived self-efficacy	Yearly gynecological care routine Personal responsibility	Personal responsibility	*Screened women:* Yearly gynecological routine
Perceived susceptibility	Recognizing risk factors of cervical cancer	Recognized the risk factors for cervical cancer	*Screened women:* Higher risk of contracting HPV or cervical cancer if they engaged in risky behaviors *Unscreened women:* would not refer to their own susceptibility but would refer to their friends’ or family’s
Perceived severity	Death due to cancer/cervical cancer Toll of cancer/cervical cancer on family Physical side-effects of cancer/cervical cancer	Recognized the toll that cancer has on the family and the severity of cervical cancer	We did not find any discordant themes
Perceived benefits	Valuing one’s health Preventing cervical cancer To be informed about health and well-being To be healthy and live longer* To encourage self-care*	Recognized overall benefits to getting screened for cervical cancer	*Screened women:* Valuing one’s health *Unscreened women:* Prevention Encouraging self-care

^*^Minor themes derived from responses from 1 to 3 participants

### Perceived barriers

Perceived barriers refer to potential difficult aspects of a particular health behavior, in this case, cervical cancer screening. These barriers might act as impediments to undertake specific actions to reduce the threat of cervical cancer [6]. Lack of time, fear of diagnosis and screening procedure, lack of priority, embarrassment, lack of information, lack of money, and having a male examiner were the most common barriers mentioned by participants in this study.

#### Lack of time

Lack of time was the barrier most commonly mentioned by participants with previous screening experience. The lack of time was due to either family or work obligations. Most of the participants who were screened and mentioned that lack of time was a barrier, said that the only reason they had been screened that day was because they had the day off and did not have to work, or had just dropped of their children at school or with someone.

“Sometimes the time, because I am working. I am studying. For example, I work Monday to Thursday from 9 to 9 at night. I have to get up at 6 in the morning, clean, make my breakfast, make lunch, leave everything ready, because I have to bring my lunch, and from Friday to Sunday I study. Friday, I leave at 4 in the morning and get back to Lima at 10 at night” – woman with previous screening experience.

#### Fear

The fear of a painful screening procedure or fear of a cancer diagnosis was a barrier identified by participants who had never been screened for cervical cancer. Participants said that they had heard from other women that getting screened is painful. The majority of participants who had never been screened said that they were afraid of being diagnosed with cancer. They recognized the severity of the disease, as they had seen how neighbors, friends, or family members had suffered. Seeing other women suffer from cancer was a deterrent to getting screened as they did not want to find themselves in the same position.

“Maybe because of the fear of knowing that there are people who have gone through this and they have ended up dead or they feel like if they fight it, it will be in vain because in the end, they are not going to have a purpose. So, it is because of doubt” – woman with no previous screening experience.

#### Lack of priority

Both groups mentioned that cervical cancer screening was not a priority in their lives. Many participants mentioned that because they are busy taking care of their families or working, their health is not a priority. Several participants mentioned that the reasons they did not get screened was because they were “lazy”. This barrier could be linked to a lack of time, the lack of information about the importance of cervical cancer screening exams or lack of prioritization of their health needs.

“Yes, but they don’t take care of themselves because they don’t want to, Miss. They don’t want to take care of themselves. They don’t love their bodies; they don’t love their health. They have to love and want their body. If you want to be okay, then you have to take care of yourself” – woman with no previous screening experience.

#### Embarrassment

Embarrassment was a barrier that was equally mentioned among the two groups. Many participants were embarrassed about going to seek care because they did not know what happened during a cervical cancer screening exam, or because they did not want to be undressed and expose their “delicate parts” to a stranger. Interestingly, embarrassment was mostly mentioned by participants who had previously been screened for cervical cancer. Many participants with previous screening experience mentioned that the original reason they delayed seeking screening was because they were embarrassed.

“I was also embarrassed, that’s why I didn’t go. That’s why I didn’t go… all of these years. I thought no, what a shame for a doctor to see me like that, oh no, that they put that thing in… I thought, me? there? Then I thought, and then I said, ‘no I have to get it done’”- woman with previous screening experience.

#### Lack of information

Lack of information was mentioned as a barrier to seeking cervical cancer twice as often among participants who had never been screened compared to those participants who had been previously screened. When participants who had never been screened talked about the lack of information, they were referring to either not knowing what happens during a screening procedure, or not knowing what cervical cancer screening is. The younger participants who had not been screened mentioned that they didn’t know they could already get screened, and they did not know that the test existed.

“one of the reasons [for not getting screened] is there is a lack of information” – woman without previous screening experience.

#### Lack of health insurance

Lack of money was mostly mentioned by participants who had been previously screened. Many said that they did not have health insurance, so they had to go to the free public clinics. However, they saw these clinics as being poorer quality, and taking a long time to get their results back. Many also mentioned that providers at these clinics were rude and were more likely to have male providers, which made women and their husbands uncomfortable.

“I think that sometimes they sincerely don’t go, because, like I have mentioned, the people in the most need here are able to access a [health insurance plan] so it doesn’t cost them. But for the people that at least have a small house, it costs us. Sometimes because one has many costs, because even a small house costs to be maintained and everything else, we deprive ourselves of many things to have something better. Even to study, we deprive ourselves from dressing better and everything else to have something more. But for people who don’t have a lot of resources, they don’t have an excuse, because they have it for free. Which is different than people like me. They have access to it for free, simply put” – woman with previous screening experience.

### Cues to action

Cues to action refers to the signals that can trigger certain actions. Cues to action for cervical cancer screening can include symptoms, environmental events, or publicity [6]. The following are cues to action that led participants to get screened, or potential cues to action for participants who have not been screened yet. For participants who have never been screened, these cues to action may be less active but are still present in a woman’s life.

#### Family

Family was an important cue to action in participants with previous screening experience. During several interviews, participants mentioned family as a trigger to seek screening. Many participants said they were getting screened because they have a family that depends on them. The most common cue to action regarding family was getting screened because women saw their role as a key caregiver for their family. They indicated this role as a reason to live longer.

“because more than anything, if you don’t want to do it for yourself- maybe when you are on your own you don’t have that stimulus- but sometimes for example, when they have kids I tell them that ‘at least do it for your kid’ I mean” – woman with previous screening experience.

Participants who had never been screened considered getting screened because they value their family. Although they have not partaken in action yet, family could be a cue to action.

“Eh, honestly it is really scary because now I have my daughter and it gives me a lot to think to be told that you have cancer and [oh my!], more than anything I think of my daughter. I mean my daughter, how is she going to be, and in this case she only has me. She doesn’t have her father anymore, she only has me and there are lot of things to think about”—woman with no previous screening experience.

#### Encouragement from family and partners

Another important cue to action was getting encouragement from a partner, mother, or other family members. Participants also mentioned that they were getting screened to set an example for their own daughters.

“I think that sometimes you need that person that is your consideration – I mean husband, kids, siblings, I don’t know, that give you that little push, so you lose fear, right?”—woman with previous screening experience.

Much like participants who had been previously screened, participants who had never been screened reported considering partaking in such behaviors because they had seen friends/family/neighbors suffer from cancer and recognized that there is a way to prevent it. They also mentioned being encouraged by a family member/partner/friend to get screened.

“…you ask, and they (women) say that they (providers) can hurt you…and sometimes they (women) respond that yes it hurts. But my aunts that I have, that are older, maybe I ask them and they say ‘no daughter, that doesn’t hurt’ they say, ‘they do it slowly’ and when they tell you that…you are encouraged to do it.” – woman with no previous screening experience.

#### Experiencing gynecological symptoms

One of the most common cues to action was experiencing gynecological symptoms not necessarily related to cervical cancer but that caused concern among women experiencing them. Several women mentioned that they had experienced “weird pains” or irregular periods and wanted to get checked. Participants who had been screened described that the reason why some women might not get screened is because they have not experienced any problems.

“My friends don’t have (health) problems, so what is the point of getting screened?” – woman with previous screening experience.

#### Recommendation by providers

Other cues to action for screening included being told to get screened in sex education classes, getting recommendations from providers due to previous medical issues or pregnancy, and physically seeing the COU near their homes.

### Perceived self-efficacy

Self-efficacy refers to the belief that a person can execute a behavior required to prevent a disease or condition even when there are barriers that need to be overcome [6]. Women with high levels of self-efficacy are more likely to engage in cervical cancer screening procedures. Participants who had been previously screened have higher self-efficacy as they have already engaged with the health behavior. Participants in both groups recognized that getting screened is something that they “have to do” because it is important for their health. Participants who had been screened usually referred to their yearly routine of getting screened in their decision-making process, whereas participants who had never been screened had a harder time articulating how they were going to engage in screening procedures.

“I am just going to have to do [the screening tests], nothing more” – woman with no previous screening experience.

“You have to do it every year, it’s normal” – woman with previous screening experience.

### Perceived severity

Perceived severity refers to feelings about the seriousness of a disease or condition, or the physical and social consequences of leaving this condition untreated [6]. For participants who had been screened and participants who had never been screened, perceived severity was similarly high, but different factors played into their decision of getting screened. The words most commonly used when participants referred to cancer or cervical cancer were: “highly dangerous”, “silent”, “terrible”, “suffering”, “depression”, “scary”, “terminal”, “damaging”, “nervous”.

Participants in both groups recognized the burden that cervical cancer can put on an individual and her family. Many participants described a friend or family member who had cancer, and the toll it took on their families.

“If you have [cancer], say goodbye to your family members” – woman with no previous screening experience.

“She has lost weight, and her – she can’t eat, her appetite has left her, she is bleeding, I don’t know, she vomits…” – woman with previous screening experience.

Participants in both groups also recognized the physical implications and symptoms that go with cancer, thus adding to its perception of severity. Although cervical cancer is preventable and treatable, late diagnosis makes the prognosis worse. Participants reported that cervical cancer is a “process” in which you start feeling bad, get diagnosed, get treatment and die. All participants indicated that cancer is not an easily treatable disease but recognized it can be treated or cured.

### Perceived susceptibility

Perceived susceptibility refers to one’s belief of the likelihood of getting a disease or a condition. If a woman thinks she is not at risk for contracting cervical cancer, she is less likely to get screened. Participants recognized some of the risk factors that might increase a woman’s chance of developing cervical cancer [6]. Specifically, participants who had been screened recognized that their risk of developing cervical cancer or contracting HPV might be higher if they had engaged in risky behaviors. Participants who had not been previously screened did not talk about their own perceived susceptibility or provide personal examples, but did talk about their friends’ and family’s risks.

“I think that most [women] take their sexual life very lightly, they don’t take care of themselves. They can get involved with whoever without knowing what diseases that person might have” – woman with previous screening experience.

### Perceived benefits

Perceived benefits refer to the acknowledgments of the severity and personal susceptibility of a disease but recognize that there are benefits to taking action to reduce the threat. Actions taken to reduce the threat have to be beneficial for the decision-maker [6]. Participants recognized overall benefits of getting screened for cervical cancer. Even participants who had never been screened before indicated that getting screened for cervical cancer has an overall positive impact on their health and wellbeing. Perceived benefits included: to be healthy, to be informed about personal health and well-being, to prevent cancer, to get health information and to live longer.

For participants who had previously been screened, valuing one’s health was reported to be an important benefit. Participants recognized that they have to get screened because their health depends on it. Throughout the interview, participants mentioned the benefits of getting screened and the risks of not getting screened. Valuing self-care can stem from fear because these participants had seen family members or neighbors suffer from cancer and recognized the importance of not going through that experience themselves. Self-care can also come from feeling susceptible to getting cervical cancer and perceiving it as severe.

“Yes, I am still, as they say, I am young right? And I still have that thing that can give me cancer, right? So, I have to be here, getting screened, I have to get examined”—woman with previous screening experience.

Two participants, one from each group, mentioned living longer as a benefit to getting screened. One participant in the never-screened group mentioned that getting screened would encourage self-care and would motivate her to get screened routinely. All 12 participants who had never been screened before mentioned benefits, compared to only eight participants in the previously screened group. Most participants who had never been screened mentioned preventing cancer as a benefit to getting screened, whereas participants who had already been screened did not really mention prevention as a benefit.

“It is for your own good, that it is for your health, that no one will return your health, not your job, not your partner if he tells you not to, nobody is going to give you back your health” – woman with previous screening experience

“If I detect that I have something early on, I can fight the cancer. Another [benefit], if it turns out I don’t have a sickness, it will help me take a bit more care for myself, with my family, with my kids, right?” – woman with previous screening experience

“The benefit is that we can still – we can still beat cancer. If it is at the beginning, we can beat cancer. We can beat cancer by wanting. You can beat cancer, it is possible” – woman with no previous screening experience

### Misconceptions

Multiple misconceptions were brought up by both groups of participants. These misconceptions fell into three categories: HPV transmission, cervical cancer understanding, and Pap smear understanding. The most common misconception among both groups was that Pap smears “wake up” the cancer. The most common misconceptions among participants who had never been screened is that when providers do a Pap smear, they rip a piece of skin out and that you can get HPV from having bad hygiene. Other misconceptions mentioned by 1 to 3 participants are summarized in Table 4.

**Table 4:** Misconceptions related to cervical cancer screening

Misconception categories	Misconceptions
HPV transmission	Can get HPV from hanging laundry to dry in an environment where there is a lot of dirt
	Can get HPV from a bathroom
	Are more prone to infections, like HPV, because you are standing for long periods of time
	Urinary infections can lead to HPV and cervical cancer
	Having bad personal hygiene can lead to HPV and cervical cancer
	Only get HPV if you have more than one partner
Cervical cancer	Cervical cancer involves tying the fallopian tubes
	Everyone is born with cancer; it’s just a matter of when you will develop it
	There is no cure for cancer
Pap smear	A pap smear can “wake up” the cancer
	A pap smear requires providers to remove a small piece of skin from the cervix
	A pap smear is a pregnancy test
	A pap smear can identify different types of gynecological conditions or cancers
	A pap smear is for removing IUDs

### Pathway to cervical cancer screening

**Fig. 1:**
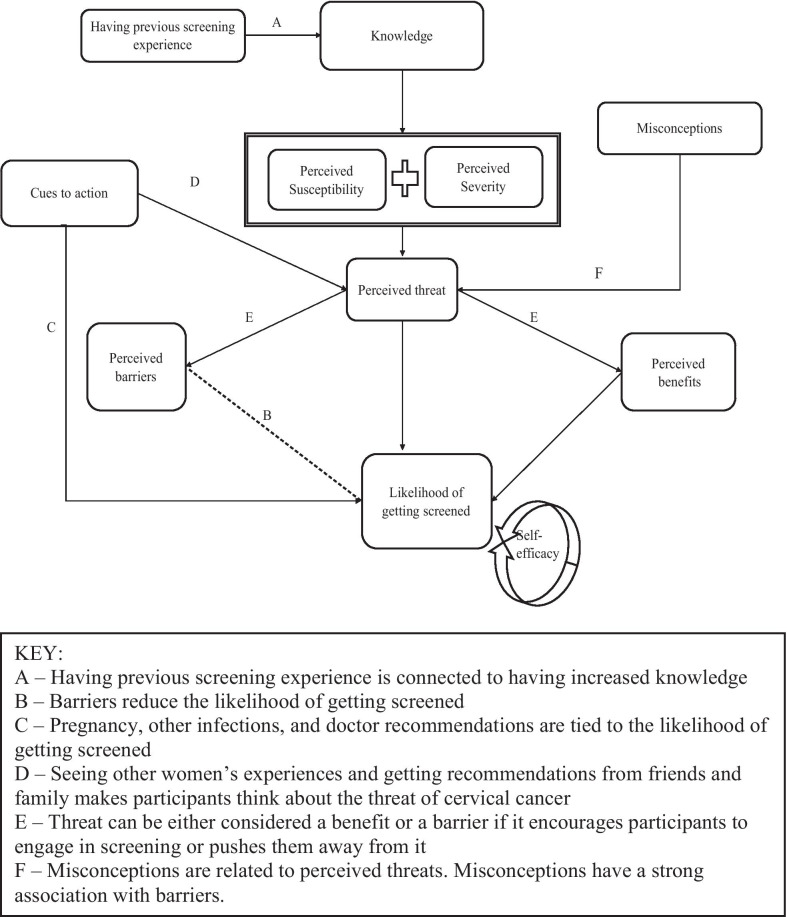
Constructs of health belief model in the context of cervical cancer screening behaviors

Based on the results, Fig. 1 depicts how we posit the Health Belief Model and its constructs interact with one another. We propose that there is a direct pathway between knowledge of HPV and cervical cancer, perceived threat of cervical cancer, perceived barriers to or benefits of screening, and the likelihood of getting screened. As shown in the figure, knowledge about HPV and cervical cancer informs the perceived threat of cervical cancer; higher perceived threat of the disease increases the likelihood of getting screened. Cues to action, as well as misconceptions, can change the threat perception. Women who perceive a high threat of cervical cancer and recognize the benefits of screening as a prevention tool, are more likely to engage in screening behaviors. Conversely, women with low perceived threat of the disease and/or recognize perceived barriers to screening are less likely to engage in screening behaviors. This proposed path is consistent with the original HBM, which suggests that a person’s perceived threat combined with their belief on the recommended course of action to reduce the threat predicts the likelihood of that person adopting said behavior [6].

## Discussion

This study explored women’s knowledge, attitudes, and practices regarding cervical cancer screening using the Health Belief Model as a framework to describe and explain why some women engage in screening procedures and why some women do not. Our findings suggest that women with previous cervical cancer screening experience are more knowledgeable than women who have not engaged in the behavior. Understanding the differences between these two groups can help future interventions target women who are not getting screened. Encouraging women who have been previously screened to continue to this practice, and to stimulate women who have never been screened to engage in this life-saving practice, can increase rates of screening uptake in Peruvian women and reduce mortality from this disease. Maintaining high uptake of screening is especially important in a setting like Peru where cervical cancer is the leading cause of death among women of reproductive age [2].

Considering that knowledge about cervical cancer and Pap smears was higher among those participants who had previously engaged in screening procedures, increasing education around health promotion regarding Pap smears could increase uptake of screening procedures. However, it is also possible that the increase in knowledge comes from the experience of getting screened and counseled.

Participants in this study mentioned their lack of health insurance and lack of time as barriers to accessing screening. A study carried out with disadvantaged communities in the United States showed that the lack of health insurance and not having a regular source of primary health care are major barriers to screening [17]. Among uninsured women, lack of time was also identified a as barrier to accessing screening [18].

The main barriers to accessing cervical cancer screening in this study were a lack of time, fear of the procedure and diagnosis, and embarrassment. These results are concordant with other studies conducted in Peru exploring women’s knowledge on cervical cancer and Pap smears [19].

Participants from both groups mentioned misconceptions they held about HPV transmission and cervical cancer equally. These misconceptions that exist can prevent women from seeking Pap smears. However, a study conducted in 2010 by Paz-Soldán et al., demonstrated that only women who had never been screened spread misconceptions based on their fears instead of actual experience with the procedure [19]. A common theme among participants in this study was a lack of understanding about prevention, as many participants thought that if there is nothing wrong with their health, there is no reason to visit a physician. These results are similar to Bingham et al.’s results from a study in Mexico where women said that they would only visit a physician if they already had developed concerning symptoms [20].

Addressing common misconceptions could also increase cervical cancer screening uptake among women who have never been screened. Many participants mentioned during their interview that if more health messaging was available on what a screening session looks like, what the requirements are to get screened, and the benefits of screening, it would encourage more women from the community to seek screening. A similar study conducted in urban areas in Lima found that creative ways to advertise about disease prevention would attract more women to take initiative on their health, including having campaigns where famous women who have had cervical cancer share their story [5]. In this present study, many participants mentioned that one of the reasons they had attended screening that day was because a famous Mexican actress had just died of ovarian cancer and that prompted them to seek care because “if she can get cancer, then [they all] can”. Influence from family, friends and partners was an important cue to action mentioned by participants. A study carried out in Zambia found that social interactions, such as that of a mom and her daughter, are an important factor for the practice of cervical cancer prevention. In this study, women who were screened for cervical cancer were more likely to vaccinate their daughters against HPV [21]. Involving the direct social network around a woman could be a potential area for intervention development.

Perceived benefits of getting screened reported by women in this study included valuing self-care, prevention of disease, and being informed about their personal health and well-being. The current study’s results are similar to those from a study conducted by Agurto et al., where Latin American participants mentioned that they had gotten screened because screening gave women “peace of mind” when they got their results back and because they felt like they were in control of their life [22].

All women in this study recognized that screening for cervical cancer was necessary. Despite this recognition, there was a missing cue to action or a barrier that outweighed the benefit for those women with no previous screening experience. Interventions directed toward the reduction of those barriers or the facilitation of a cue to action could increase women’s cervical cancer screening uptake.

### Strengths

The strengths of this study include the use of in-depth interviews to capture the richness of participants’ experience in a way that avoided pre-determined assumptions of the researcher, which often happens when using surveys alone. Another strength of the study was having two different groups that spoke to the personal experiences of women who had been screened for cervical cancer and women who had never been screened.

### Limitations

This study has some limitations. First, due to the qualitative nature of the study and small sample size (n = 24), results cannot be generalized to all Peruvian women. Second, the survey items used to assess knowledge, attitudes, and practices have not been validated. The classification of participants as low, fair or high knowledge has not been rigorously tested; we used these classifications in this study descriptively only. In fact, screening itself may be associated with knowledge improvement and a score like this could be used to measure those differences. Also, because we did not hold constant variables such as economic status in recruiting participants for the two groups, it is possible that differences between women who had and had not been screened relate to factors other than those reported in the results. Lastly, using the HBM has some limitations as well. The model is more descriptive than explanatory, and it does not provide a strategy for changing health behaviors. The individual constructs are useful, but the model should be paired with other models that can account for environmental context and suggest strategies for change [19].

## Conclusions

Lack of knowledge about cervical cancer screening procedures among women who have never been screened was found in metropolitan Lima. Poor knowledge and practices among this group of participants is an important barrier that must be addressed. Understanding knowledge, attitudes, and practices among women who have and have never engaged in cervical cancer screening procedures using the Health Belief Model can be an effective way of exploring the barriers women face when getting screened, as well as understanding the cues to action that lead to women getting screened. Future studies should look at what knowledge women get from cervical cancer screening and ways that the interaction with the provider encourages women to keep getting screened for cervical cancer. Studying ways interventions can be effective to target women who have never been screened is also important research. However, even with increased screening, there are other bottlenecks that should be addressed, such as access to providers, low awareness of the importance of screening, and failure of providers to recommend Pap smears.

## Supplementary Information

**Additional file 1. S1**. Supplementary methods.** S2**. KAP Survey.** S3**. In-depth interview guide.** S4**. Demographic characteristics of study participants (n = 24).

## Data Availability

The datasets generated and/or analyzed during the current study are not publicly available but are available from the corresponding author on reasonable request.

## Abbreviations

HPV: Human Papilloma Virus
La Liga: La Liga Contra el Cancer – Peru
HBM: Health Belief Model
