# Supplementary material for: Knowledge, attitudes, and practices regarding cervical cancer screening among women in metropolitan Lima, Peru: a cross-sectional study
Source: BMC Womens Health. 2021 Aug 18;21:304. doi: 10.1186/s12905-021-01431-0 (PMC8371792; doi:10.1186/s12905-021-01431-0)

**ADDITIONAL FILES**

**Additional file 1: Supplementary methods**

Each COU is comprised of a midwife who performs the screening and a nurse technician who supports the midwife by doing administrative tasks, data entry, and triage. COUs are set up close to busy locations (e.g., markets, schools, public parks, municipalities) in the districts. Screening is offered between 9 am – 2:30 pm every weekday for one to two-week time periods per community; after a few weeks’ absence, the COU returns for another one-two weeks. On average, each COU screens about 30 women per day.

The study was conducted in metropolitan Lima, Peru. Metropolitan Lima is divided into 50 districts; 43 districts belong to Lima Province and 7 are part of the Constitutional Province of Callao (Callao). For this study, participants were recruited from four districts. Three of the study locations belonged to Lima Province (Surquillo, Ate, and Villa el Salvador) and one study location (Ventanilla) was part of Callao. These districts were chosen for the study because the COU’s were scheduled to provide screening services there during the study period.

Cards with response options were given to the participants to guide them through the multiple-choice questions. Responses were recorded on a paper form and later double-entered electronically using the Qualtrics^XM^ survey platform.

In-depth interviews were audio-recorded on encrypted-devices to facilitate transcription and translation to English. Field notes were taken during the interview by a research assistant or the local study coordinator, in order to capture salient points mentioned during the interview. After the interview, MP met with the research assistant or local study coordinator to debrief and identify interview challenges or additional domains to explore in subsequent interviews.

The instruments were initially drafted in English. Once finalized, they were translated into Spanish and revised by the local study coordinator to ensure that vocabulary and phrasing of the questions made sense for the Peruvian context. The structured survey and interview guide were pilot tested with staff from *La Liga* and updated before starting data collection.

Audio-recordings of interviews were transcribed and translated into English by both MP and EC.

MP has a B.A. in Anthropology. She is trained in ethnographic and qualitative data collection. At the time of the study MP was a Candidate for a Master of Science in Global Health.

**Additional Files 2: KAP Survey**

**DEMOGRAPHIC INFORMATION**

1. What is the highest level of school you have completed or the highest degree you received?
   - None
   - Unfinished primary school
   - Finished primary school
   - Unfinished secondary school
   - Finished secondary school
   - Unfinished higher non-university
   - Finished higher non-university
   - Unfinished higher university
   - Finished higher university
2. Which of the following best describes your marital status?
   - Single 🡪 skip to question 4
   - Married/in union
   - Divorced/separated/widow
3. How old were you when you got married?
   - Free response
4. How many children have you given birth until now?
   - free response 🡪 if 0, skip to question 6
5. How old were you when you had your first child?
   - Free response
6. How many people live with you in your house? Please include yourself in the count.
   - 1-20 free response
7. Which of the following categories best describes you current employment status?
   - Currently employed
   - Recently unemployed
   - Have not been employed in the last 12 months
   - Other (specify) _____________________
   - **Never been employed**
8. In the past 4 weeks, did you worry that your household would not have enough food?
   - No
   - Yes 🡪 skip to 8a

8a. How often did this happen?

- - - - Rarely
      - Sometimes
      - Often

1. In the past four weeks, were you or any household member not able to eat the kinds of foods you preferred because of a lack of resources?
   - No
   - Yes 🡪 skip to 9a

9a. How often did this happen?

- - - - Rarely
      - Sometimes
      - Often

1. In the past four weeks, did you or any household member have to eat a limited variety of foods due to a lack of resources?
   - No
   - Yes 🡪 skip to 10a

10a. How often did this happen?

- - - - Rarely
      - Sometimes
      - Often

1. In the past four weeks, did you or any household member have to eat some foods that you really did not want to eat because of a lack of resources to obtain other types of food?
   - No
   - Yes 🡪 skip to 11a

11a. How often did this happen?

- - - - Rarely
      - Sometimes
      - Often

1. In the past four weeks, did you or any household member have to eat a smaller meal than you felt you needed because there was not enough food?
   - No
   - Yes 🡪 skip to 12a

12a. How often did this happen?

- - - - Rarely
      - Sometimes
      - Often

1. In the past four weeks, did you or any household member have to eat fewer meals in a day because there was not enough food?
   - No
   - Yes 🡪 skip to 13a

13a. How often did this happen?

- - - - Rarely
      - Sometimes
      - Often

1. In the past four weeks, was there ever no food to eat of any kind in your household because of lack of resources to get food?
   - No
   - Yes 🡪 skip to 14a

14a. How often did this happen?

- - - - Rarely
      - Sometimes
      - Often

1. In the past four weeks, did you or any household member go to sleep at night hungry because there was not enough food?
   - No
   - Yes 🡪 skip to 15a

15a. How often did this happen?

- - - - Rarely
      - Sometimes
      - Often

1. In the past four weeks, did you or any household member go a whole day and night without eating anything because there was not enough food?
   - No
   - Yes 🡪 skip to 16a

16a. How often did this happen?

- - - - Rarely
      - Sometimes
      - Often

Thank you. In the next set of questions, I will ask you questions about what you know regarding cervical cancer.

Do you have any questions before we proceed? [PAUSE FOR QUESTIONS]

**KNOWLDEGE ON CERVICAL CANCER**

I will read a list of words. For each, please say if you know what it is, if you have heard the word but don’t know what it is or if you haven’t heard of it. Ready?

17. Cervical cancer

- - - I know what it is
    - I’ve heard of it but don’t really know what it is
    - I haven’t heard of it
    - REFUSED

18. Cervical cancer screening

- - - I know what it is
    - I’ve heard of it but don’t really know what it is
    - I haven’t heard of it
    - REFUSED

19. Female reproductive cancer

- - - I know what it is
    - I’ve heard of it but don’t really know what it is
    - I haven’t heard of it
    - REFUSED

20.Papanicolaou test, also called a Pap smear

- - - I know what it is
    - I’ve heard of it but don’t really know what it is
    - I haven’t heard of it
    - REFUSED

21. Human Papilloma Virus, also known as HPV

- - - I know what it is
    - I’ve heard of it but don’t really know what it is
    - I haven’t heard of it
    - REFUSED

22. Visual inspection with acetic acid, also known as VIA

- - - I know what it is
    - I’ve heard of it but don’t really know what it is
    - I haven’t heard of it
    - REFUSED

23. Colposcopy

- - - I know what it is
    - I’ve heard of it but don’t really know what it is
    - I haven’t heard of it
    - REFUSED

24. HPV Vaccine

- - - I know what it is
    - I’ve heard of it but don’t really know what it is
    - I haven’t heard of it
    - REFUSED

**CERVICAL CANCER SCREENING**

1. How often do you think you need a Pap test to screen for cervical cancer?
   - Every year
   - Every 6 months
   - Every 3 years
   - Every 5 years
   - DON’T KNOW
   - REFUSED
2. What is the recommended age for when a woman should start getting screened?
   - 18
   - 21
   - 30
   - 40
   - 50
   - 60
   - Other
   - DON’T KNOW
   - REFUSED
3. Have you heard of other tests for cervical cancer screening?
   - Yes
   - No
   - REFUSED

27a. If yes, which ones? [MARK ALL THAT APPLY]

- - - - - Visual inspection
        - Liquid-based cytology
        - HPV DNA testing

Please tell me if you agree or disagree with the following statements

1. Menstrual bleeding between periods should be considered normal
   - Agree
   - Disagree
   - DON’T KNOW
   - REFUSED
2. A woman should bear her first child by age 20
   - Agree
   - Disagree
   - DON’T KNOW
   - REFUSED
3. Women with more sexual partners are predisposed to cervical cancer
   - Agree
   - Disagree
   - DON’T KNOW
   - REFUSED
4. Women should get internal exams at least every 3 years
   - Agree
   - Disagree
   - DON’T KNOW
   - REFUSED
5. If any lady in the neighborhood is suffering from cervical cancer, I should stay away from her
   - Agree
   - Disagree
   - DON’T KNOW
   - REFUSED
6. All women should be screened for cervical cancer
   - Agree
   - Disagree
   - DON’T KNOW
   - REFUSED
7. Cervical cancer can be prevented
   - Agree
   - Disagree
   - DON’T KNOW
   - REFUSED
8. Cervical cancer can be treated
   - Agree
   - Disagree
   - DON’T KNOW
   - REFUSED

**RISK FACTORS FOR CERVICAL CANCER**

Are the following risk factors for cervical cancer? Answer “yes” “no” or “don’t know”

1. Family history of cervical cancer
   - - Yes
     - No
     - DON’T KNOW
     - REFUSED
2. Using a intrauterine device, meaning the birth control IUD
   - - Yes
     - No
     - DON’T KNOW
     - REFUSED
3. Having HIV
   - - Yes
     - No
     - DON’T KNOW
     - REFUSED
4. Not using condoms
   - - Yes
     - No
     - DON’T KNOW
     - REFUSED
5. Poor menstrual hygiene
   - - Yes
     - No
     - DON’T KNOW
     - REFUSED
6. Sexually transmitted infections
   - - Yes
     - No
     - DON’T KNOW
     - REFUSED
7. Smoking
   - - Yes
     - No
     - DON’T KNOW
     - REFUSED
8. Sexual activity at early age
   - - Yes
     - No
     - DON’T KNOW
     - REFUSED
9. Multiple sexual partners
   - - Yes
     - No
     - DON’T KNOW
     - REFUSED
10. Multiple pregnancies
    - - Yes
      - No
      - DON’T KNOW
      - REFUSED
11. Use of birth control pills for more than 5 years.
    - - Yes
      - No
      - DON’T KNOW
      - REFUSED
12. By what methods can cervical cancer be prevented? [MARK ALL THAT APPLY]
    - It can’t be prevented
    - Sexual abstinence
    - Being faithful to partner
    - Using condoms
    - Vaccination
    - Other: _____
    - DON’T KNOW
    - REFUSED

Human Papilloma Virus (HPV) is a viral infection. Please answer true or false about the following statements about HPV

1. Having HPV increases your risk of cervical cancer
   - True
   - False
   - DON’T KNOW
   - REFUSED
2. HPV is spread by sexual contact
   - True
   - False
   - DON’T KNOW
   - REFUSED
3. People with HPV always have vaginal symptoms
   - True
   - False
   - DON’T KNOW
   - REFUSED

Please answer “YES” “NO” or “don’t know” to the following statements.

1. Are all women of childbearing age at risk for cervical cancer?
   - - Yes
     - No
     - DON’T KNOW
     - REFUSED
2. Are women with gynecological problems at risk for cervical cancer?
   - - Yes
     - No
     - DON’T KNOW
     - REFUSED
3. Are pregnant women at risk for cervical cancer?
   - - Yes
     - No
     - DON’T KNOW
     - REFUSED
4. Are sexually active women at risk for cervical cancer?
   - - Yes
     - No
     - DON’T KNOW
     - REFUSED

**SYMPTOMS OF CERVICAL CANCER**

Which of the following are symptoms of cervical cancer? Please say “yes” “no” or “don’t know”.

1. Foul smelling vaginal discharge
   - - Yes
     - No
     - DON’T KNOW
     - REFUSED
2. Bleeding after sex
   - - Yes
     - No
     - DON’T KNOW
     - REFUSED
3. Postmenopausal bleeding
   - - Yes
     - No
     - DON’T KNOW
     - REFUSED
4. Abdominal pain
   - - Yes
     - No
     - DON’T KNOW
     - REFUSED

**Additional Files 3: In-depth interview guide**

**OPEN ENDED QUESTIONS FOR THOSE WHO HAVE BEEN SCREENED**

Thank you for answering the questions so far. In order to understand more about this topic of cervical cancer, I would like to ask you about your experiences and the experiences of other women who have been screened for cervical cancer. There are no right or wrong answers to the questions I am about to ask you. You are the expert of your experiences. I will ask you some questions that will guide you but would like for you to do more of the talking. Do you have any questions before I proceed?

1. You mentioned that you have been screened for cervical cancer before. Think about the most recent time when you were screened/[IF RECRUITED FROM MOBILE UNIT] Think about your screening visit today. Could you describe the screening visit to me?

*{INTENT: This intent of this question is to describe positive screening behaviors, and understand if there are any experiences with screening which may encourage or discourage women from coming back}*

- 1. What made it good or bad?
  2. How did it differ from any previous experiences you may have had being screened?

1. What made you decide to get screened for cervical cancer? *(cues to action)*

*{INTENT: The intent of this question is to capture factors impacting the decision-making process that specifically translate to the women being screened.}*

- 1. Was there someone/something that influenced you to come (friend, family member, heard something on the radio, read about it, community outreach, doctor)?

1. When you think of cervical cancer, what are some things that come to mind? (*Perceived seriousness/perceived susceptibility)*

{*INTENT: The intent of this questions is to examine how women think of themselves at risk of cervical cancer and how serious they believe cervical cancer is*}

- 1. Can you tell me about any personal experiences you may have had with cervical cancer, for example, with a family member or friend who may have been diagnosed?
     1. How did being diagnosed with cervical cancer affect their life and their family’s life?
  2. As a reminder, you can decline to answer a question if it makes your uncomfortable. How do you think your risk of cervical cancer compares to other women?
  3. How does your knowledge of other cancers/cancer in general, influence what you think about cervical cancer?
  4. When you think about your risk of cancer, how does that affect whether or not you get screened?

1. What are some of the benefits of being screened for cervical cancer? *(perceived benefits)
   {INTENT: The intent of this question is to assess what women see as benefits of being screened for cervical cancer and assess if these benefits encourage screening behaviors}*
   1. If you were to tell your friends about reasons to get screened, what would you tell them?
2. What things do your friends and family do that you think can put them at risk of cervical cancer? *(perceived threat)*
   {*INTENT: The intent of this question is to examine what are some factors that women see as threats to their health or the health of others}*
   1. What kinds of things lead to people developing cervical cancer?
   2. What would you tell your family or friends who are engaging in these risky behaviors?
3. For some women, getting screened is a difficult thing and some women do not feel like they have confidence or the ability to do this. Why do you think this is the case? *(self-efficacy)*
   *{INTENT: The intent of this question examines what makes women feel comfortable to make the decision to get screened and if their confidence influences their decision}*
   1. What contributed to you feeling comfortable to come for screening? What made you feel confident that you made a good choice?
4. What are some things that make it hard for you to come and get screened? *(perceived barriers)
   {INTENT: the intent of this question is to examine what barriers women face when they want to get screened}*
   1. What makes it easy for you to come here?
   2. When you hear from your friends or family, what do they mention that makes it hard for them to get screened?
   3. If you know of someone who does not get screened, why is that? Why do you think some people don’t come? (childcare, transportation, money, access)
5. We are trying to better understand what women know about the services that La Liga provides.
   {*INTENT: the intent of the question is to help La Liga examine the reach they have in their community}*
   1. How did you find out about the screening that La Liga does?
   2. What services does La Liga offer that makes it easy to get screened?
6. We are interested in learning what goes into a woman’s decision-making process of getting screened for cervical cancer again in the future.
   {*INTENT: the intent of the question is to understand what influences a woman to get screened again and what aids in their decision}*
   1. Do you think you’ll get screened for cervical cancer again?
   2. How will you decide when to be screened again?

Thank you. This brings us to the end of the interview. I really appreciate the time you have spent and the experiences and thoughts you have shared with me today. Do you have any questions before we end? [PAUSE FOR QUESTIONS]. Thank you.

**OPEN ENDED QUESTIONS FOR THOSE WHO HAVE NOT BEEN SCREENED**

Thank you for answering the questions so far. In order to understand more about this topic of cervical cancer, I would like to ask you about your decisions regarding cervical cancer screening and the decision of other women who do not get screened for cervical cancer. There are no right or wrong answers to the questions I am about to ask you. You are the expert of your experiences. I will ask you some questions that will guide you but would like for you to do more of the talking. Do you have any questions before I proceed?

1. You mentioned that you have never been screened for cervical cancer before. What are some reasons why you have never been screened? *(perceived barriers)
   {INTENT: the intent of this question is to examine what barriers exist and how they influence a woman’s decision to get screened}*
   1. Is there someone/something that influenced you to not go for screening (friend, family member, heard something on the radio, read about it, community outreach, doctor)
   2. What reasons have you heard from other family members or friends for not getting screened?
2. Did you ever contemplate going for screening? If no, why not? If yes, what ultimately aided in your decision not to get screened?  *(cues to action)
   {INTENT: the intent of this question is to explore what factors influence a woman’s decision to not get screened}*
   1. If you had the opportunity to be screened, what would you need in order to make the decision (information, support) in favor of being screened?
   2. What would incentivize/motivate you to get screened?
3. When you think of cervical cancer what are some things that come to mind? (*Perceived seriousness/perceived susceptibility)
   {INTENT: The intent of this questions is to examine how women think of themselves at risk of cervical cancer and how serious they believe cervical cancer is}*
   1. Can you tell me about any personal experiences you may have had with cervical cancer, for example, with a close family member or friend who may have been diagnosed?
      1. How did being diagnosed with cervical cancer affect their own life and their family life?
   2. As a reminder, you can decline to answer a question if it makes your uncomfortable. How do you think your risk of cervical cancer compares to other women? Does your answer affect your decision to get screened or not?
   3. When you think of cervical cancer, do you also think about other types of cancer? How do you think they might be similar?
4. According to you, what would be some of the benefits of getting screened? *(perceived benefits)
   {INTENT: The intent of this question is to assess what women see as benefits of getting screened for cervical cancer and assess if these benefits discourage screening behaviors}*
   1. Why do you think some women get screened?
   2. If you have been recommended to not get screened, what have those recommendations looked like? What do your family and friends think are benefits of not getting screened?
5. What things do your friends and family do that you think can put them at risk of cervical cancer/cancer in general? *(perceived threat)*
   *{INTENT: The intent of this question is to examine what are some factors that women see as threats to their health or the health of others around them}*
   1. What do people think contributes to getting cancer?
   2. What would you tell your family or friends who are engaging in these risky behaviors?
6. For some women, getting cancer screening can feel difficult and some women do not feel like they have confidence or the ability to do this. Why do you think this is the case? *(self-efficacy)*
   {INTENT: The intent of this question is to examine whether confidence in one self is an influencing factor on is whether or not women get screened}
   1. How much did a lack of confidence or ability to get screened contribute to your decision?
   2. What would help you build some of that confidence or sense of ability to get screened?
7. We are trying to better understand what women know about the services that La Liga provides. Can you tell me about what La Liga does and who they are?
   {*INTENT: The intent of the question is to help La Liga examine what groups they should be targeting and how to update their outreach strategy}*
   1. What could La Liga do to reach women like you about cancer screening?
   2. How do you get information about events and services in the community?
8. Do you think you will get screened for cervical cancer in the future?

Thank you. This brings us to the end of the interview. I really appreciate the time you have spent and the experiences and thoughts you have shared with me today. Do you have any questions before we end? [PAUSE FOR QUESTIONS]. Thank you

**Additional file 4: Demographic characteristics of study participants (n=24)**

| **Variable** | **Previously screened**  **(n=12)**  **n (%)** | **Never screened**  **(n=12)**  **n (%)** | **Total**  **(n=24)**  **n (%)** |
| --- | --- | --- | --- |
| **Age** |  |  |  |
| 18-20 years | 0 (0) | 3 (25) | 3 (12.5) |
| 21-29 years | 4 (33.3) | 5 (41.7) | 9 (37.5) |
| 30-39 years | 4 (33.3) | 1 (8.3) | 5 (20.8) |
| 40-49 years | 3 (25) | 2 (16.7) | 5 (20.8) |
| 50-64 years | 1 (8.3) | 1 (8.3) | 2 (8.3) |
| **Schooling** |  |  |  |
| Did not finish primary school | 0 (0) | 2 (16.7) | 2 (8.3) |
| Did not finish secondary school | 4 (33.3) | 0 (0) | 4 (16.7) |
| Finished secondary school | 1 (8.3) | 1 (8.3) | 2 (8.3) |
| Did not finish higher non-university | 1 (8.3) | 6 (50) | 7 (29.2) |
| Finished higher non-university | 3 (25) | 1 (8.3) | 4 (16.7) |
| Did not finish higher university | 2 (16.7) | 2 (16.7) | 4 (16.7) |
| Finished higher university | 1 (8.3) | 0 (0) | 1 (4.2) |
| **Marital status** |  |  |  |
| Single | 0 (0) | 6 (50) | 6 (25) |
| Married/In union | 11 (91.7) | 5 (41.7) | 16 (66.7) |
| Divorced/separated/widow | 1 (8.3) | 1 (8.3) | 2 (8.3) |
| **Employment status**  Currently employed  Have not been employed in the last  12 months  Recently unemployed | 5 (41.7)  5 (41.7)  2 (16.7) | 1 (8.3)  5 (41.7)  6 (50) | 6 (25)  10 (41.7)  8 (33.3) |
| **Average age at first marriage (in years) *** | 20 [15-37] | 19 [18-30] | 20 [15-37] |
| **Average number of births*** | 2 [0-5] | 1 [0-6] | 2 [0-6] |
| **Average age at first child (in years) *** | 19 [17-33] | 19 [16-33] | 19 [16-33] |

**Data presented as median [range]*


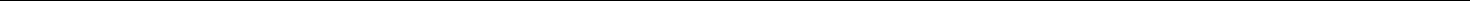

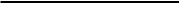

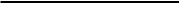

Supplement: Supplementary file 1 — Additional file 1. S1. Supplementary methods. S2. KAP Survey. S3. In-depth interview guide. S4. Demographic characteristics of study participants (n = 24). [file 12905_2021_1431_MOESM1_ESM.docx]
